# A rare case of disseminated cysticercosis

**DOI:** 10.1186/1471-2334-14-S3-P71

**Published:** 2014-05-27

**Authors:** S Praneetha, R Priyanka, D Srilekha

**Affiliations:** 1 Melmaruvathur Adhiparasakthi Institute of Medical Sciences & Research, Melmaruvathur-603319, India

## Background

Neurocysticercosis is a common tropical parasitic disease of the CNS. But the disseminated form is an uncommon manifestation of this disease. Here we report a case of disseminated cysticercosis.

## Case report

A 10-year-old girl was brought to the hospital with complaints of fever, headache and vomiting for 10days duration before a month. Fever was high grade, associated with chills. There was no history of seizures. Immunized upto date with normal development. On examination, the child was dull looking with regular pulse rate of 100/min. CNS examination showed mild neck stiffness, otherwise unremarkable. Peripheral smear study showed microcytic hypochromic anemia with mild eosinophilia. Fundus examination was normal. Child was admitted and administered antibiotics after which she recovered. Fifteen days after discharge she presented with history of diplopia and headache. There was history of beef intake for 3 years and the recent intake was before 2 months. Eye examination showed well defined white lesion of 0.5cm in the anterior chamber of eye that moved with head movement. Fundus examination showed mild papilledema. Non contrast CT brain was near normal. MRI brain findings showed multiple hyperintense lesions of less than 3mm on T2WI which appears hypointense on T1WI in the gangliocapsular, thalamus and subcortical regions. A diagnosis of cysticercosis was made and the ocular cyst had to be surgically removed and treatment with prednisolone 1-2mg/day had to be started 2-3 days prior and continued with cysticidal therapy - Albendazole 15mg/kg/day for 5 to 28days. Patient asked to follow up after cyst removal for management and is asymptomatic presently.

## Conclusion

Maintaining personal hygiene and avoiding undercooked meat could limit cysticercosis.

